# Selection of an optimal macrocyclic chelator improves the imaging of prostate cancer using cobalt-labeled GRPR antagonist RM26

**DOI:** 10.1038/s41598-019-52914-y

**Published:** 2019-11-19

**Authors:** Bogdan Mitran, Helge Thisgaard, Sara Rinne, Johan Hygum Dam, Frishta Azami, Vladimir Tolmachev, Anna Orlova, Ulrika Rosenström

**Affiliations:** 1 Department of Medicinal Chemistry, Uppsala University, Uppsala, Sweden; 2 PET & Cyclotron Unit, Department of Nuclear Medicine, Odense University Hospital, Odense, Denmark; 3 Department of Clinical Research, University of Southern Denmark, Odense, Denmark; 4 Science for Life Laboratory, Department of Medicinal Chemistry, Uppsala University, Uppsala, Sweden; 5 Department of Immunology, Genetics and Pathology, Uppsala University, Uppsala, Sweden

**Keywords:** Positron-emission tomography, Prostate cancer, Cancer imaging

## Abstract

Gastrin-releasing peptide receptors (GRPRs) are promising targets in oligometastatic prostate cancer. We have recently used ^55^Co (T_1/2 = _17.5 h) as a label for next day PET imaging of GRPR expression obtaining high imaging contrast. The radionuclide-chelator combination can significantly influence the biodistribution of radiopeptides. Therefore, in this study, we hypothesized that the properties of ^55^Co-labeled PEG_2_-RM26 can be improved by identifying the optimal macrocyclic chelator. All analogues (X-PEG_2_-RM26, X = NOTA,NODAGA,DOTA,DOTAGA) were successfully labeled with radiocobalt with high yields and demonstrated high stability. The radiopeptides bound specifically and with picomolar affinity to GRPR and their cellular processing was characterized by low internalization. The best binding capacity was found for DOTA-PEG_2_-RM26. *Ex vivo* biodistribution in PC-3 xenografted mice was characterized by rapid blood clearance via renal excretion. Tumor uptake was similar for all conjugates at 3 h pi, exceeding the uptake in all other organs. Higher kidney uptake and longer retention were associated with N-terminal negative charge (DOTAGA-containing conjugate). Tumor-to-organ ratios increased over time for all constructs, although significant chelator-dependent differences were observed. Concordant with affinity measurements, DOTA-analog had the best retention of activity in tumors, resulting in the highest tumor-to-blood ratio 24 h pi, which translated into high contrast PET/CT imaging (using ^55^Co).

## Introduction

Prostate cancer is a highly heterogeneous, multifactorial disease with a complex underlying biology that evolves from initial tumorigenesis to metastatic spread and castration-resistance. During prostate cancer progression, tumors become more invasive, acquiring properties for increased metastatic potential, becoming more difficult to treat. There are no curative therapies for late stage prostate cancer and treatments are focused primarily on disease control and improving the quality of life.

In recent years, the concept of oligometastatic prostate cancer has been receiving increasing attention. Oligometastatic disease is defined as an intermediate state in prostate cancer progression characterized by limited metastatic spread^1^. It is hypothesized that this intermediate state has a different biology compared to widespread metastatic disease and is susceptible to treatments designed to influence the rate of progression, and potentially even cure the patients^2^. Therefore, diagnostic tools that can identify oligometastatic prostate cancer and stratify the patients for personalized treatment strategies are highly required.

Currently, prostate cancer diagnostic relies mainly on PSA screening and biopsies based methods, which have significant limitations. PSA screening has limited diagnostic specificity and cannot distinguish between prostate cancer and normal conditions that are associated with high PSA level^3^. Biopsies are invasive, have a high sampling error of up to 40%, and can lead to severe complications^4^. Moreover, these diagnostic modalities cannot differentiate between local, regional, and distant disease, which is a prerequisite for oligometastatic prostate cancer diagnosis.

These major limitations of the main prostate cancer diagnostic tools place molecular imaging in a unique position where it can significantly contribute not only in the initial diagnosis and patient stratification, but also in monitoring the response to therapy. However, despite its significant potential, molecular imaging of oligometastatic prostate cancer is not without challenges, and the highest sensitivity and specificity are required for imaging of small metastases^5^.

Imaging of oligometastatic prostate cancer requires a molecular target that is predominantly expressed in earlier stages of disease such as gastrin releasing peptide receptor (GRPR). GRPR overexpression was documented in primary tumors (63–100%), lymph node metastases (86%) and bone metastases (53%)^6,7^. Moreover, normal prostate tissue as well as benign prostate tumors are mostly GRPR-negative^8^.

Bombesin (BN), a small linear peptide, binds to GRPRs with high affinity and selectivity. The use of peptides such as bombesin-based ligands for tumor targeting offers the advantages of excellent vascular permeability and rapid access to tumors. Consequently, several bombesin derivatives have been developed in the past decades. Encouraged by the success reported for somatostatin antagonists^9^, the initial consensus favoring bombesin agonists has also shifted towards the use of potent bombesin antagonists^10^.

We have previously reported on an antagonistic analogue of bombesin NOTA-PEG_2_-RM26 labeled with ^111^In and ^68^Ga that had a favorable biodistribution profile with a high and specific GRPR-mediated tumor uptake^11^. Interestingly, the fast clearance of radioactivity from blood and normal tissues in combination with long retention in tumors resulted in a pronounced increase in tumor-to-background ratios over time up to 24 h for ^111^In-NOTA-PEG_2_-RM26. This data indicates that a long lived radionuclide would be desirable for high contrast imaging.

The use of positron emission tomography (PET) would additionally improve imaging of low abdominal lymph node involvement which requires the highest possible sensitivity. In addition to higher sensitivity, PET also provides better spatial resolution and more accurate quantification compared to single photon emission computed tomography (SPECT). Among the possible long lived positron-emitting radiometals, ^55^Co (T_1/2_ = 17.5 h) has a high positron abundance (76% β^+^) and good ratio between annihilation photons and co-emitted gammas. Cobalt-55 can be produced on low-energy biomedical cyclotrons at most PET centers and/or be distributed to distant sites. Production costs for cobalt-55 are comparable with copper-64. Interestingly, the use of radiocobalt for labeling of NOTA-PEG_2_-RM26 has led to a remarkable increase in tumor-to-organ ratios than the previously evaluated ^68^Ga and ^111^In-labeled NOTA-PEG_2_-RM26^12^. Tumor-to-blood ratio for ^55/57^Co-NOTA-PEG_2_-RM26 at the 3 h time point was twofold higher than ^111^In- and 4-fold higher compared to ^68^Ga-labeled analogues. Moreover, exceptionally high tumor-to blood, tumor-to-pancreas, tumor-to-stomach, and tumor-to kidney ratios were achieved at the later time point of 24 h post-injection (pi)^12^.

The remarkable potential of ^55^Co-NOTA-PEG_2_-RM26 for high contrast PET imaging of GRPR-expressing tumors, raise the question of whether the imaging properties of this conjugate can be further improved. Changes of chelating moieties are likely to provide this opportunity since it is well known that the structure and physical properties of radionuclide-chelator complex can have a large impact on the biodistribution and targeting properties of radiopeptides^11,13–16^.

**Figure 1:**
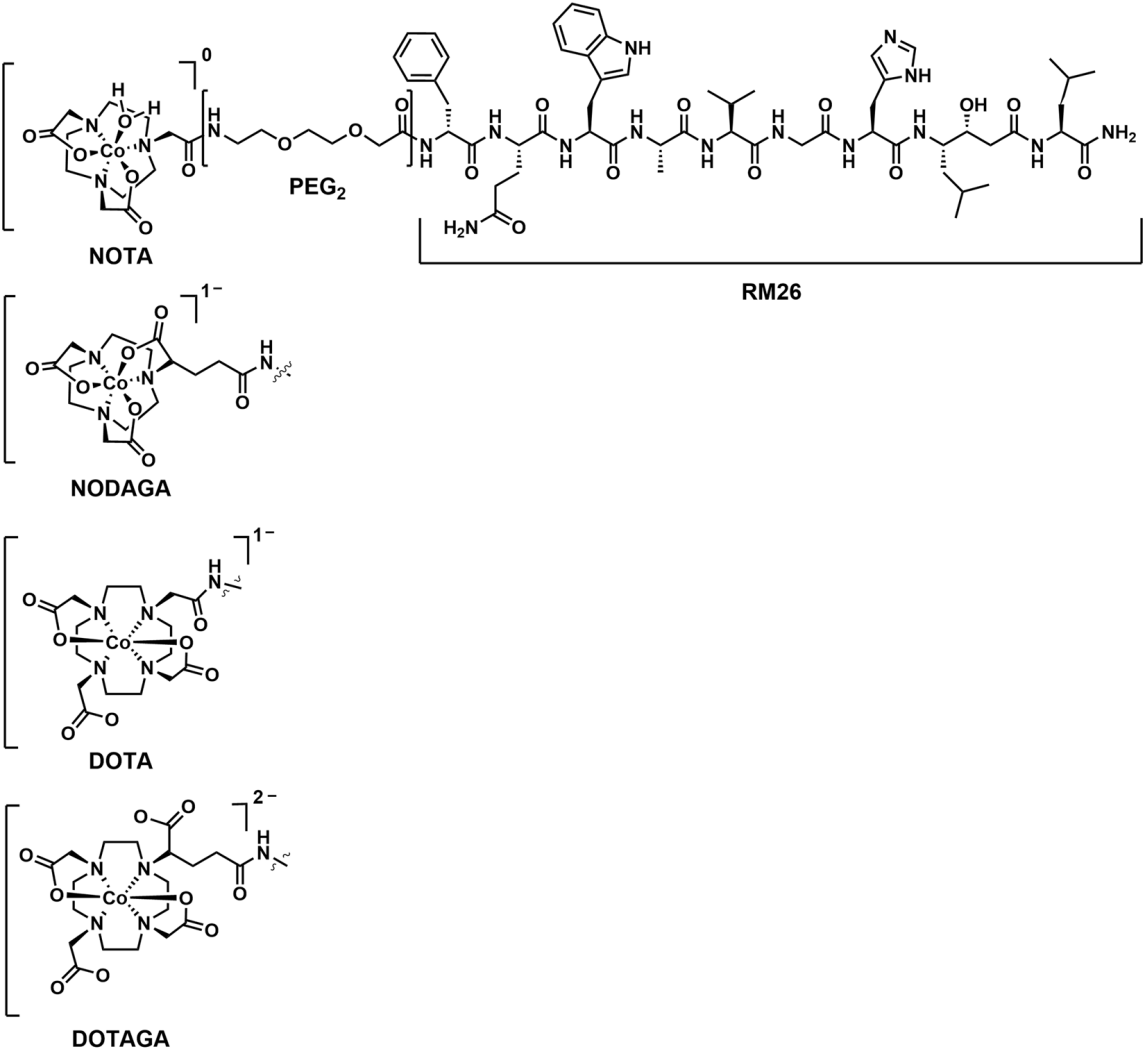
Schematic overview of the structure of different chelators: NOTA, NODAGA, DOTA and DOTAGA, coupled to RM26 peptide via a PEG_2_ spacer.

The goal of this study was to further improve the biodistribution of radiocobalt-labeled RM26. We have coupled tri- and tetra-aza macrocyclic chelators to the N-terminus of PEG_2_-RM26 (X-PEG_2_-RM26, X = NOTA, NODAGA, DOTA, DOTAGA, Fig. 1) and investigated the respective conjugates for their efficacy in imaging of GRPR-expressing tumors. Cobalt-57 (T_1/2_ = 271.6 d, E = 122 keV (86%), 136 keV (10%)) was used for *in vitro* and *ex vivo* experiments for convenience as proposed earlier^12^.

## Results

### Labeling and stability

All conjugates were successfully labeled with ^57^Co and ^55^Co with yields exceeding 98% (Table 1). Stability was evaluated for all labeled constructs after incubation in the presence of 1000-fold molar excess of EDTA for 1 h at RT. The radionuclide release was lower than 0.4% for all analogues, as shown by ITLC analysis (Table 1).

**Table 1:** Labeling and complex stability of radiocobalt labeled X-PEG_2_-RM26 (X = NOTA, NODAGA, DOTA and DOTAGA).

^55/57^Co-X-PEG_2_-RM26	NOTA	NODAGA	DOTA	DOTAGA
Labeling yield for ^55^Co, %	98.5	98.8	99.3	99.7
Retention time, min	8.91	8.93	8.71	8.73
Retention factor	3.24	3.25	3.15	3.16
Labeling yield for ^57^Co, %	99.9 ± 0.1	99.0 ± 0.2	99.5 ± 0.1	99.5 ± 0.2
Release of ^57^Co in the presence of excess EDTA	0.4 ± 0.3	0.3 ± 0.2	0.3 ± 0.1	0.4 ± 0.2

Release of cobalt from peptide bound complex was checked after 1 h incubation at room temperature in the presence of 1000 excess of EDTA. Data are presented as average ± standard deviation.

### *In vitro* studies

**Figure 2:**
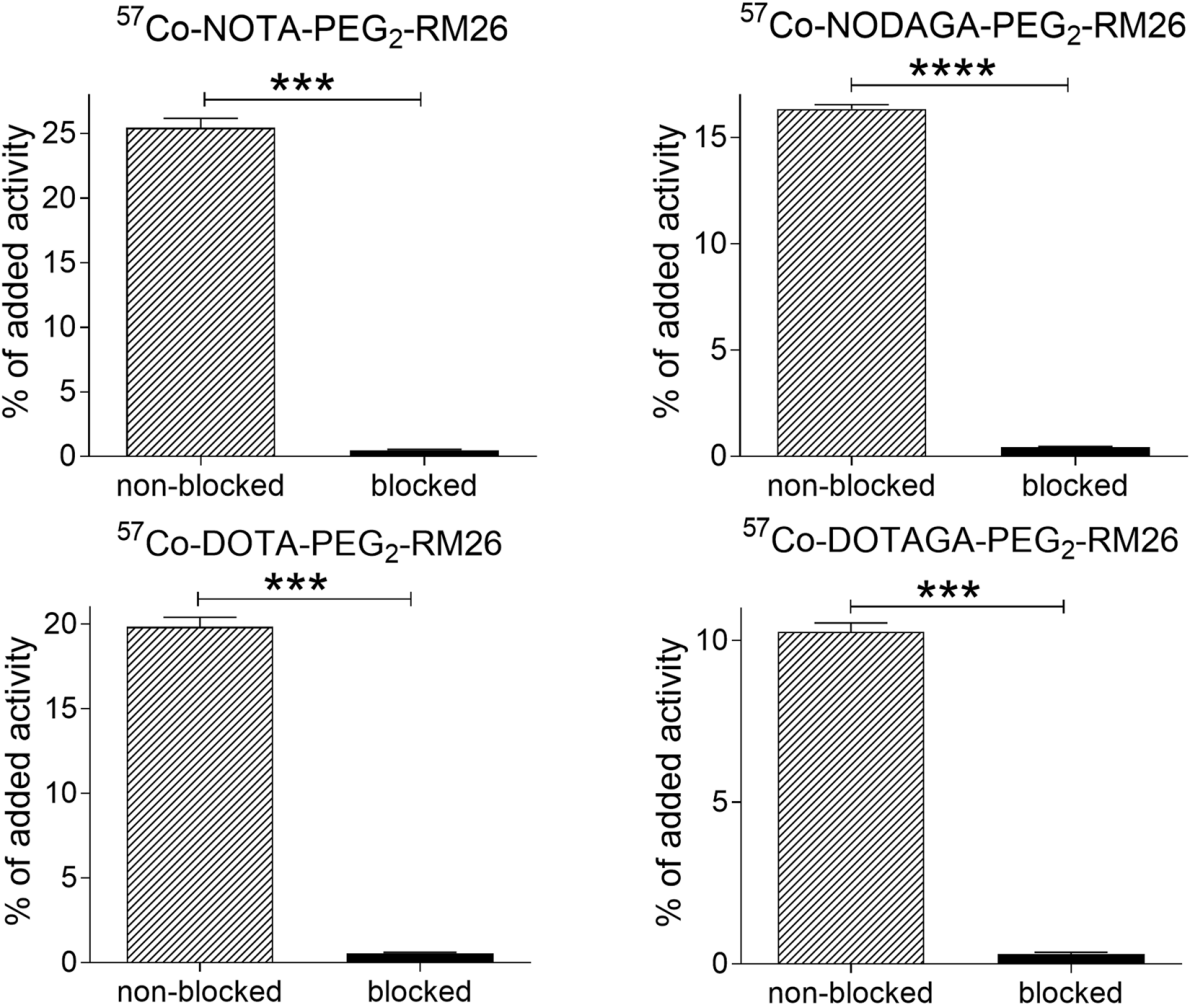
Binding specificity of ^57^Co-X-PEG_2_-RM26 (X = NOTA, NODAGA, DOTA and DOTAGA) to GRPR-expressing PC-3 cells. Two groups of culture dishes containing PC-3 cells were incubated with radiolabeled conjugates (1 nM). One set of dishes was pre-saturated with excess amount of non-labeled conjugates (blocked). Data are presented as average ± standard deviation. Data were analyzed by an unpaired, two-tailed t-test.

The results of the *in vitro* binding specificity test are presented in Fig. 2. Pre-saturation of receptors with excess amount of non-labeled conjugates caused a significant reduction of ^57^Co-X-PEG_2_-RM26 uptake. This demonstrated that the uptake was receptor mediated.

**Figure 3:**
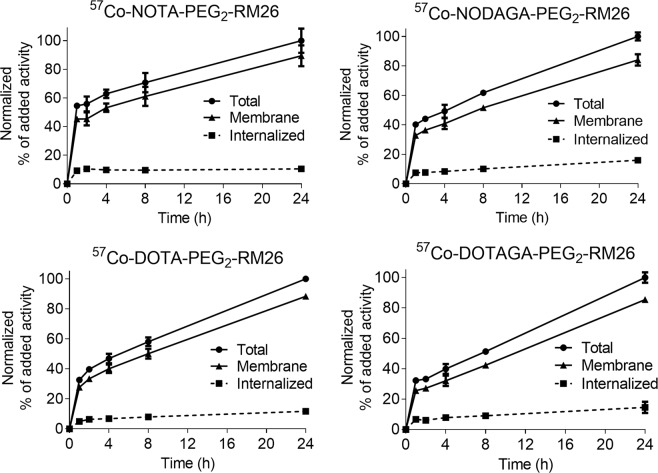
Binding and cellular processing of ^57^Co-X-PEG_2_-RM26 by GRPR-expressing PC-3 cells. Radiolabeled conjugates were incubated up to 24 h at 37 °C. Cell-bound activity was normalized to the maximum uptake. Data are presented as mean value ± standard deviation. Error bars may not be visible because they are smaller than point symbols.

Data concerning cellular processing and internalization of ^57^Co-X-PEG_2_-RM26 are presented in Fig. 3. The internalization pattern was similar for all analogues reaching only 10–12% of cell-associated activity after 24 h incubation. The binding pattern was characterized by a rapid increase of total cell-associated radioactivity within the first hour of incubation, followed by a gradual increase up to 24 h for all conjugates.

**Figure 4:**
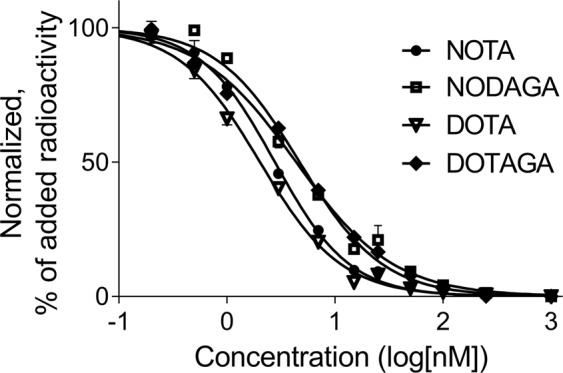
Inhibition of ^125^I-Tyr^4^-BBN binding to PC-3 cells with ^nat^Co-X-PEG_2_-RM26 [X = NOTA (●), NODAGA (■), DOTA (▼) and DOTAGA (◆)]. Data are presented as mean value ± standard deviation.

The half-maximal inhibitory concentration (IC_50_) was evaluated using ^125^I-Tyr^4^-BBN as displacement radioligand (Fig. 4, Table 2). All IC_50_ values were in the low nanomolar range. The best binding affinity was observed for DOTA and NOTA-containing analogues. Higher IC_50_ values, corresponding to lower affinity were obtained for NODAGA and DOTAGA-containing constructs.

**Table 2:** Inhibition of ^125^I-Tyr^4^-BBN binding to PC-3 cells with ^nat^Co-X-PEG_2_-RM26 (X=NOTA, NODAGA, DOTA and DOTAGA).

^57^Co-X-PEG_2_-RM26	Competitive binding assay - IC_50_ (nM)^a^	Real time binding kinetics measurement^b^		
		*k*_*a*_ *(M*^−1^*s^*−1*^)	*k*_*d*_ *(s*^*−1*^)	*K*_*D*_ *(pM)*
NOTA	2.8 ± 0.2	(1.9 ± 0.5) x 10^5^	(1 ± 0.2) x 10^–5^	56 ± 3
NODAGA	4.7 ± 0.7	(1.7 ± 0.2) x 10^5^	(1 ± 0.5) x 10^−5^	66 ± 25
DOTA	2.0 ± 0.2	(3 ± 0.3) x 10^5^	(9.9 ± 3.4) x 10^−6^	33 ± 12
DOTAGA	4.4 ± 0.4	(1.9 ± 0.2) x 10^5^	(3.2 ± 0.3) x 10^−5^	167 ± 22

^a^Data are presented as the mean values of three dishes ± SD. ^b^Data are presented as average ± SD.

The binding affinity of ^57^Co-X-PEG_2_-RM26 to PC-3 cells was also assessed in real-time using LigandTracer Yellow instruments (Table 2). Similar to IC_50_ results, ^57^Co-DOTA-PEG_2_-RM26 had the best affinity, with a rapid binding and a slow dissociation, followed by ^57^Co-NOTA-PEG_2_-RM26. Worse K_D_ values were obtained for NODAGA and DOTAGA-containing analogues. It must be noted that all K_D_ values were in the low picomolar range. By measuring the time to equilibrium using real-time kinetic binding studies, the risk of premature interruption of incubation is eliminated. This is very important in the characterization of high affinity binders that require extremely long time to reach equilibrium. Therefore, the binding affinity can be severely underestimated in end-point assays (IC_50_ values differ in three orders of magnitude compared to K_D_ values for the studied conjugates).

### *In vivo* studies

Biodistribution of free cobalt was studied 3 and 24 h pi (Supplementary Fig. 1). Uniform activity distribution was observed at both time points with somewhat elevated uptake in liver that was in good agreement with previously published data^17^.

**Figure 5:**
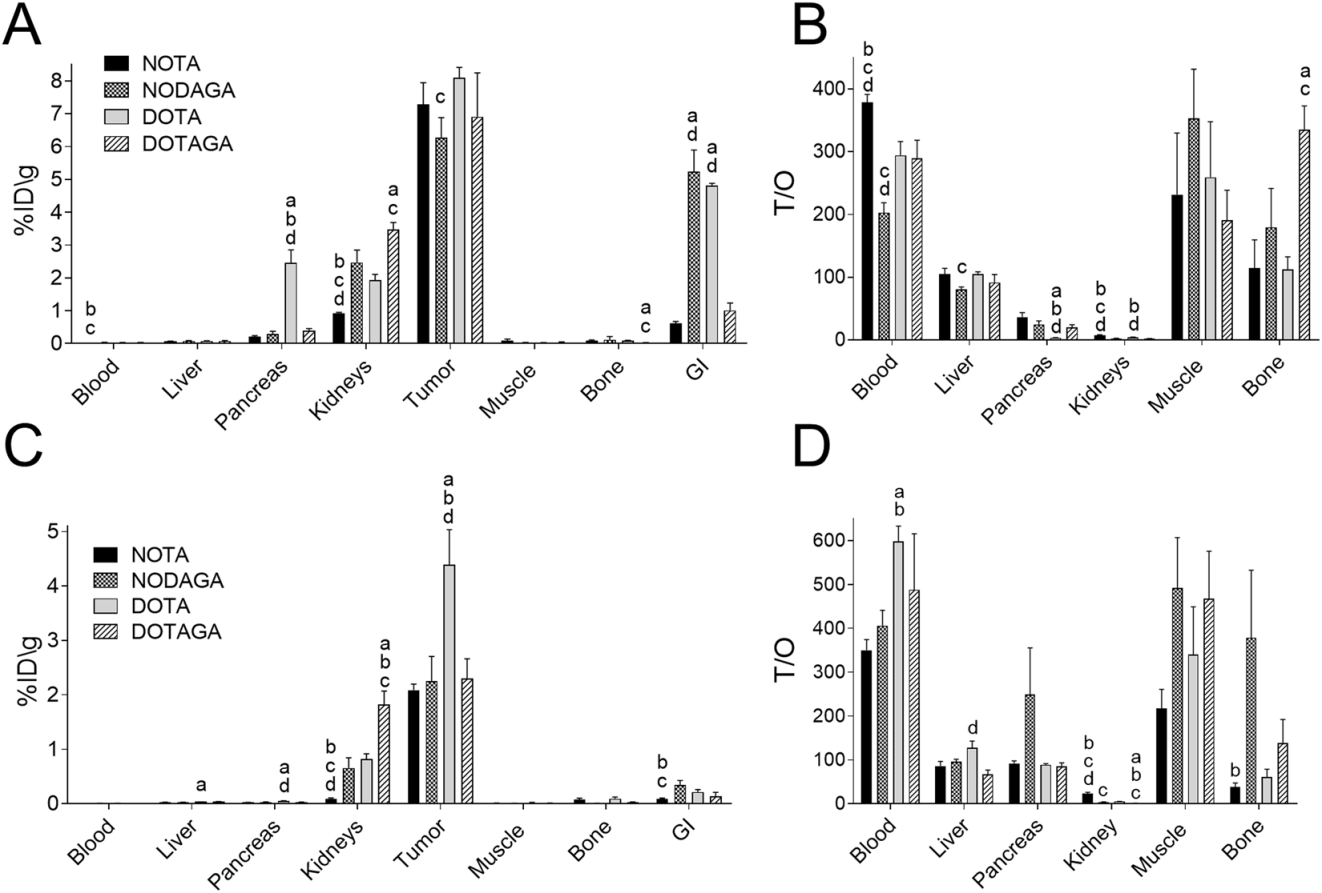
Biodistribution of ^57^Co-X-PEG_2_-RM26 (X = NOTA, NODAGA, DOTA and DOTAGA) in PC-3 xenografted BALB/C nu/nu mice (**A**) 3 h and (**C**) 24 h pi. Tumor-to-normal-tissue ratios (**B**) 3 h and (**D**) 24 h pi. Data were analyzed by one-way ANOVA with Bonferroni correction for multiple comparisons. Significant difference (p < 0.05) at the same time point. a. Significantly different from NOTA b. Significantly different from NODAGA c. Significantly different from DOTA d. Significantly different from DOTAGA.

Biodistribution of radiocobalt-labeled conjugates and comparison of their *in vivo* tumor targeting was evaluated in BALB/c nu/nu mice bearing PC-3 xenografts 3 and 24 h pi (Fig. 5). The common feature of all conjugates was the rapid clearance from blood and normal tissues. Tumor uptake was similar for all analogues 3 h pi, exceeding the uptake in all normal organs, including excretory organs. Despite the similar initial uptake, radioactivity retention in tumors differed and 24 h pi tumor radioactivity was twofold higher for the DOTA-containing conjugate (4.4 ± 1.3%IA/g for DOTA, 2.1 ± 0.2%IA/g for NOTA, 2.3 ± 0.9%IA/g for NODAGA, and 2.3 ± 0.7%IA/g for DOTAGA). The accumulated activity in GRPR-expressing organs was also significantly higher for ^57^Co-DOTA-PEG_2_-RM26, with more than 6-fold higher pancreatic uptake compared to all other constructs. The main excretion pathway was through the kidneys for all analogs, although significant differences in renal uptake and retention were observed. ^57^Co-NOTA-PEG_2_-RM26 had a significantly lower kidney uptake both 3 h and 24 h pi, followed by NODAGA and DOTA-containing constructs. The highest renal activity uptake and the longest activity retention were observed for the DOTAGA-construct. The differences in kidney uptake became more pronounced at the later time point (24 h pi) with DOTAGA-conjugate having a 20-fold higher uptake compared to NOTA- and a twofold higher uptake compared to NODAGA- and DOTA- containing analogues. Interestingly, a high uptake was detected for NODAGA and DOTA-carrying radioligands in the GI tract with contents (~5%ID for NODAGA and DOTA vs ≤ 1%ID/g for NOTA and DOTAGA, 3 h pi).

Tumor-to-organ ratios are presented in Fig. 5. NOTA-coupled analogue provided significantly higher tumor-to-blood and tumor-to-kidney ratios 3 h pi. However, at the later time point, the better retention in tumor of ^57^Co-DOTA-PEG_2_-RM26 resulted in a twofold higher tumor-to-blood ratio compared to NOTA-coupled analogue.

*In vitro* binding specificity to GRPR for ^57^Co-DOTA-PEG_2_-RM26 was confirmed in NMRI mice (Supplementary Fig. 2). Significant decrease in activity uptake was found in pancreas and tissues of GI tract (organs with GRPR expression) when higher peptide dose was injected.

### Imaging

**Figure 6:**
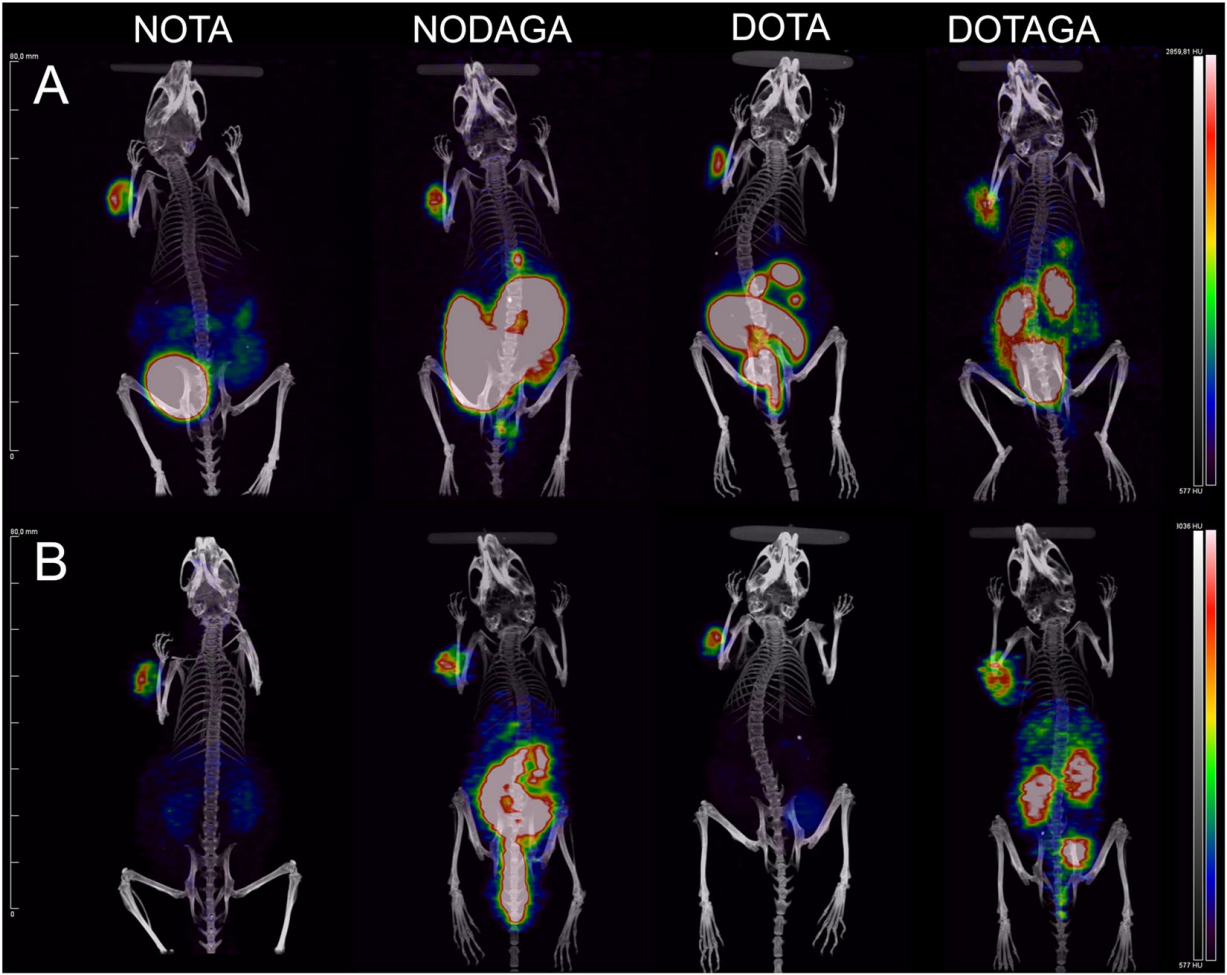
Coronal MIP PET/CT images showing tracer distribution in PC-3 xenografted NOD-SCID mice. The animals were injected with 0.3 nmol (range: 0.24–0.32 nmol, approx. 2.5 MBq) ^55^Co-X-PEG_2_-RM26 (X = NOTA, NODAGA, DOTA and DOTAGA).

GRPR expression in PC-3 xenografted mice was successfully visualized using PET/CT after the intravenous injection of ^55^Co-DOTA-PEG_2_-RM26 (X = NOTA, NODAGA, DOTA, DOTAGA) (Fig. 6). The activity uptake in the GI tract for NODAGA and DOTA-carrying radioligands and the high kidney uptake of the DOTAGA-conjugate can be clearly visualized on the coronal MIP images acquired 3 h pi. At the later time-point of 24 h, the faster clearance of radioactivity from normal organs compared to GRPR-expressing tumors resulted in high contrast imaging. The best contrast 24 h pi was obtained for DOTA and NOTA-containing analogues.

## Discussion

Receptors for small regulatory peptides are often overexpressed in human tumors. One example of such receptors are GRPRs, which are overexpressed in early prostate cancer and can be targeted for diagnostic imaging and therapy of oligometastatic prostate cancer. Peptides such as the bombesin-based ligands targeting GRPRs are attractive for radionuclide molecular imaging due to their relatively small size which translates into fast blood clearance, fast tumor penetration and inexpensive production.

In this study, we have attempted to optimize the GRPR antagonist PEG_2_-RM26 by identifying the most suitable macrocyclic chelator for labeling with radiocobalt (^55/57^Co-X-PEG_2_-RM26, X = NOTA, NODAGA, DOTA, DOTAGA). Earlier data have shown that chelators had a profound influence on the biodistribution profile of the ^68^Ga and ^111^In-labeled X-PEG_2_-RM26 significantly altering the blood clearance, uptake in tumors and GRPR-expressing organs and clearance of activity from kidneys^15,16^. The four macrocyclic chelators differ in size, with NOTA and NODAGA containing three nitrogen donor atoms compared to the four nitrogens of DOTA and DOTAGA (Fig. 1). In addition, NODAGA is a derivative of NOTA and DOTAGA is a DOTA-derivative each containing an additional carboxylic acid group.

Two characteristics of the radiometal-chelator complex have been previously identified to have major impact on the behavior of radiopeptides: complex charge and geometry. The charge of the complex depends on the valency of the metal and the number of available carboxylic acid arms of the chelator. Labeling of analogues with radiocobalt is performed in acidic conditions that prevents oxidation of Co^2+^ by atmospheric oxygen to Co^3+^. Heppeler *et al*. have previously shown Co^II^ to be stable against oxidation during labeling and complexation reactions of DOTA-containing somatostatin analogs^13^. Consequently, the overall charge of the Co^2+^-chelator complex is different compared to the previously evaluated ^68^Ga^3+^ and ^111^In^3+^ labeled analogues and will be 0 for NOTA, −1 for NODAGA and DOTA, and −2 for DOTAGA. In terms of size, cobalt ionic radius is similar to the one of gallium (0.65 Å) and smaller than indium (0.92 Å). Similarly to gallium, Co^2+^ is hexacoordinated to NOTA and DOTA and forms DOTA complexes with distorted octahedral coordination geometry^13,18^. Structures of Co^2+^ complexes with NODAGA and DOTAGA can be deduced from the aforementioned parent NOTA and DOTA chelators.

All tested analogues were successfully labeled with radiocobalt with yields exceeding 99% for ^57^Co and 98% for ^55^Co. Labeling did not affect the binding specificity of the conjugates to GRPR despite harsh labeling conditions. In principle, different chelator-radionuclide combinations could influence the internalization rate of radiometal-labeled peptides. However, data from cellular processing assay showed a similar internalization pattern for all analogues, with a very low fraction of internalized radioactivity. Although a low internalization is common for antagonists, the internalized fraction for the radiocobalt-labeled conjugates was three fold lower compared to the same analogues labeled with other radiometals such as ^111^In and ^68^Ga^15,16^. These findings were in agreement with data published for proteins labeled with cobalt^12,19,20^ and might indicate lower residualizing properties of the radiocatabolites for the cobalt labeled conjugates.

The binding properties of ^nat^Co-X-PEG_2_-RM26 were evaluated in a competitive binding assay showing IC_50_ values in the low nanomolar range for all conjugates. Surprisingly, IC_50_ values did not follow the same pattern as previously observed for indium and gallium labeled X-PEG_2_-RM26 or for somatostatin analogues which showed that a negative N-terminal charge will decrease the binding affinity^21,22^. Although the presence of a -2 charge for DOTAGA-cobalt complex resulted in the worst binding affinity, the best binding affinity was observed for negatively charged DOTA-containing analogue, followed by neutral NOTA-construct.

Equilibrium dissociation constant (K_D_) values obtained by measuring the binding affinity in real-time were in the picomolar range and had the same pattern as the IC_50_ values with the best binding affinity observed for ^57^Co-DOTA-PEG_2_-RM26, followed by NOTA-, NODAGA- and DOTAGA-conjugates indicating that N-terminus charge is not the only factor that affects the binding affinity of radiocobalt-labeled conjugates.

Biodistribution pattern of ^57^Co-X-PEG_2_-RM26 in tumor-bearing mice was characterized by rapid clearance from blood which is important for high-contrast molecular imaging. The clearance was predominantly via kidney ultrafiltration and a low activity uptake was observed for all analogues in liver and unspecific compartments. Comparison of distribution of ^57^Co-X-PEG_2_-RM26 with distribution of free cobalt corroborated the high stability of all studied cobalt complexes. Concentration of free cobalt in blood and normal organs was one-two orders of magnitude higher than for tested conjugates. Tumor uptake was on the same level for all conjugates 3 h pi, exceeding the uptake in all normal organs, including excretory organs. Interestingly, despite similar tumor uptake, the activity accumulation in GRPR-expressing organs differed significantly between conjugates. Notably, the pancreatic uptake (the organ with the most abundant GRPR expression) was more than 6-fold higher for DOTA-containing analogue compared to all other constructs. This discrepancy between the uptake in tumor and GRPR-expressing organs was previously reported for the same conjugates labeled with ^111^In^16^ and for other radiolabeled bombesin analogues^23–25^ and is most likely due to differences in affinity towards human and murine GRPR.

A clear influence of the N-terminal charge was observed on kidney activity uptake and retention with the lowest uptake and retention for neutral NOTA- and the highest uptake and the longest retention for the -2 charged DOTAGA analogue. A surprising finding was the high uptake in GI tract (with contents) for NODAGA- and DOTA-containing analogues 3 h pi that did not correlate with conjugate lipophilicity nor complex charge and geometry. Comparison of biodistribution patterns of ^57^Co-DOTA-PEG_2_-RM26 injected in low and high peptide dose and free cobalt in NMRI mice (Fig. 5A and Supplementary Fig. 1) lead us to the conclusion that the high activity uptake in GI tract observed for these conjugates partially consists of GRPR mediated uptake in GI tract tissue (specific uptake in pancreas, stomach and intestine) and partially due to GI tract content. Because we did not observe a biodistribution pattern characteristic for free cobalt (elevated activity concentration in blood, uniform high activity uptake in normal organs, and slow whole body clearance) for the cobalt labeled peptides we concluded that the elevated activity uptake in GI tract was not the result of complex instability *in vivo*. With high probability the GI tract content includes products of hepatic degradation of labeled peptide, either cobalt-chelate complexes or released cobalt, indicating that some excretion occurs through the bile for these conjugates. However, liver uptake was below 0.1%ID/g for all constructs 3 h pi and activity was rapidly excreted from GI tract for DOTA conjugate.

Perhaps the most remarkable finding of this study was the two fold higher retention of activity in tumors for DOTA-containing analogue that correlates with the better binding affinity found for this analogue. Due to the faster clearance of activity from blood and normal organs compared to tumors, tumor-to-organ ratios generally improved between 3 and 24 h pi, with ^57^Co-DOTA-PEG_2_-RM26 having the highest tumor-to-blood ratio 24 h pi. PET/CT imaging using ^55^Co-X-PEG_2_-RM26 were in accordance with the biodistribution results with NOTA-containing analogue demonstrating superior imaging properties 3 h pi. However, at the later time-point of 24 h pi, the best imaging contrast was obtained for ^55^Co-DOTA-PEG_2_-RM26.

Comparing indium and cobalt labeled X-PEG_2_-RM26 conjugates 24 h pi we have found that while for indium the best contrast was achieved for neutrally charged In-NODAGA complex^16^, negatively charged Co-DOTA conjugate had the best performance for cobalt labeled conjugates. Indium and cobalt labeled NODAGA-conjugates similarly had high uptake in GRPR-expressing organs and tissue in gastrointestinal tract at early time point. This activity uptake rapidly decreased for ^111^In-NODAGA-PEG_2_-RM26, but not for the conjugate labeled with cobalt.

High contrast imaging is essential for the evaluation of intraprostatic and peri-prostatic tissues and for local staging of suspect lymph nodes in prostate cancer. This is due to the fact that almost 80% of metastatic lymph nodes have a diameter of less than 8 mm, smaller than what could be detected in CT and MRI (10 mm)^26^.

We have compared published data for tracers suitable for next day imaging of GRPR expression using PET (labeled with copper-64 (T½ 17.5 h, β^+^ 18%) and cobalt-55) and SPECT (labeled with indium-111 (T_½_ 2.6 d)) (Supplementary Table 3). Cobalt labeled DOTA-PEG_2_-RM26 conjugate has appreciably higher tumor-to-blood and tumor-to-liver ratios than conjugates labeled with copper^22,27^. Conjugates suitable for SPECT imaging generally demonstrated high tumor-to-organ ratios^16,28,29^ and together with the new generation of SPECT cameras, indium labeled GRPR antagonists should provide images with high contrast. GRPR agonists demonstrated the lowest tumor-to-organ ratios as already mentioned above^29,30^.

In conclusion, the charge and structure of chelating moiety had a profound influence on tumor targeting and biodistribution profile of radiocobalt-labeled GRPR antagonist RM26. The net charge of the radionuclide-chelator complex influenced the uptake and retention of activity in the kidneys. The highest binding affinity observed for DOTA-containing analogue resulted in better retention of radioactivity in tumors indicating that ^55^Co-DOTA-PEG_2_-RM26 is a promising candidate for next day high contrast PET imaging of GRPR-expressing tumors.

## Methods

^57^Co cobalt chloride was purchased from PerkinElmer Sweden (Upplands Vasby, Sweden). ^55^Co was produced and purified in house as previously described^31,32^. Buffers used for labeling were prepared in-house using high-quality Milli-Q water and chemicals supplied by Merck (Darmstadt, Germany). After preparation, buffers were pretreated with Chelex 100 resin (Bio-Rad Laboratories, Richmond, USA) to remove metal contamination.

The synthesis and characterization of X-PEG_2_-RM26 (RM26 = [D-Phe^6^, Sta^13^, Leu^14^]Bombesin[6–14], X = NOTA, NODAGA, DOTA and DOTAGA) (Fig. 1) were performed as previously described^11^.

The GRPR-expressing prostate cancer cell line PC-3 used in the cell studies was obtained from ATCC, LGC Promochem. Cells were cultured in RPMI-1640 media supplemented with 10% fetal calf serum (Sigma), PEST (penicillin 100 IU/mL, streptomycin 100 g/mL), and 2 mM L-glutamine (all from Biochrom AG, Berlin, Germany). This medium is referred to as complete medium in the text. The cells were detached using a trypsin-EDTA solution (0.05% trypsin, 0.02% EDTA in buffer; Biochrom AG).

An automated γ-spectrometer (3-inch NaI(Tl) detector, 2480 WIZARD^2^, PerkinElmer) was used to measure radioactivity.

### Statistics

Data were analyzed using GraphPad Prism (version 7.03, GraphPad Software Inc.) to determine significant statistical differences (p < 0.05) by an unpaired, two-tailed t-test and by one-way ANOVA with Bonferroni correction for multiple comparisons.

### Labeling and stability

Labeling of X-PEG_2_-RM26 with ^57^Co was performed using 10 nmol (aq. 1 nmol/µL) of peptide, buffered with 80 µL ammonium acetate (0.2 M, pH 5.5). For labeling with ^55^Co, X-PEG_2_-RM26 were buffered with 80 µL acetate buffer (0.2 M, pH 5.5), followed by dynamic microwave heating to 90 °C for up to 3 min in a sealed vial using a PETWave (CEM Corp.). After labeling with ^55^Co, radiochemical yield was evaluated by radio HPLC as previously described^32^. Stability was evaluated by instant thin-layer chromatography (ITLC). In this experiment, ^57^Co-labeled peptides were incubated in the presence of 1000-fold molar excess of EDTA disodium salt (Sigma) for 1 h at RT. The ITLC strips (150–771 Dark Green, Tec-Control Chromatography strips from Biodex Medical Systems) were eluted with citric acid (0.2 M, pH 2.0). In this system, free ^57/55^Co or radiocobalt chelated by EDTA migrate with the eluent front (R_f_ = 1.0) while the radiolabeled constructs remain on the application point (R_f_ = 0.0). The system was previously validated by radio-HPLC and sodium dodecyl sulfate polyacrylamide gel electrophoresis (SDS-PAGE).

### *In vitro* studies

GRPR-binding specificity of the X-PEG_2_-RM26 variants after ^57^Co-labeling was evaluated on PC-3 cells as previously described^16^. Briefly, PC-3 cells (800,000 per dish) were incubated with 1 nM ^57^Co-X-PEG_2_-RM26 for 1 h at 37 °C under 5% CO_2_. One group of Petri dishes was pre-saturated with 200-fold excess of unlabeled peptide added 10 min prior to the addition of radiolabeled compounds. After incubation, cells were washed with serum-free media and were detached using 0.5 mL trypsin-EDTA solution. Cell-associated radioactivity was measured in an automated gamma-counter and presented as percentage from added radioactivity.

For internalization studies, PC-3 cells (800,000 per dish) were incubated with ^57^Co-X-PEG_2_-RM26 (2 nM) at 37 °C under 5% CO_2_. At predetermined time points (1, 2, 4, 8, and 24 h after incubation start), the incubation medium was removed and the cells were treated using the previously described acid wash method in order to collect the membrane-bound and internalized radioactivity^11^.

The half-inhibitory concentration (IC_50_) was estimated for all the ^nat^Co-labeled analogues by an *in vitro* competitive binding assay using ^125^I-Tyr^4^-BBN as a displacement radioligand. Briefly, PC-3 cells were incubated with ^125^I-Tyr^4^-BBN for 5 h at 4 °C in the presence of 12 increasing concentrations of ^nat^Co-X-PEG_2_-RM26 ranging from 0 to 1000 nM. After incubation, the cells were collected and measured in an automated gamma-counter. IC_50_ values were calculated by fitting the data by nonlinear regression using GraphPad Prism software.

Kinetics of ^57^Co-X-PEG_2_-RM26 binding to and dissociation from living PC-3 cells were measured in real time using LigandTracer Yellow instruments (Ridgeview Instruments AB) at RT as previously described^33^. The uptake curves were recorded at 0.6 and 1.8 nM. Thereafter, the ^57^Co-X-PEG_2_-RM26- containing medium was replaced by fresh medium and the dissociation curve was monitored for 1000 min. The TraceDrawer Software (Ridgeview Instruments) was used to calculate the equilibrium dissociation constant (K_D_) based on the association and dissociation rates.

### *In vivo* studies

The animal experiments were planned and performed in accordance with the national regulations on laboratory animals’ protection and were approved by the Ethics Committee for Animal Research in Uppsala and Denmark, respectively. Euthanasia was performed by intraperitoneal injection of Ketalar-Rompun solution (10 mg/mL Ketalar and 1 mg/mL Rompun; 20 µL/g body weight) and all efforts were made to minimize suffering. Groups of 4 mice per data point were used.

The biodistribution of cobalt-57 chloride (pre-incubated with ammonium acetate buffer (0.2 M, pH 5.5)) was studied in NMRI mice 3 and 24 h pi (25 kBq in 100 µL PBS per mouse). Blood samples were collected by heart puncture. Lung, liver, spleen, pancreas, stomach, small intestines, kidneys, muscle, bone, and the rest of gastrointestinal tract with its content were collected, weighed and measured in a gamma-counter. The organ uptake values were expressed as a percentage of injected dose per gram of tissue weight (%ID/g) with the exception of gastrointestinal tract and the remaining carcass, which were calculated as %ID per whole sample.

The biodistribution and targeting properties of ^57^Co-X-PEG_2_-RM26 were evaluated in female BALB/c nu/nu mice (weight: 18 ± 2 g). GRPR-expressing xenografts were established by subcutaneous injection of 8 × 10^6^ PC-3 cells/mouse, 2 weeks prior to the experiment. At the time of experiment, the mice were intravenously injected with ^57^Co-X-PEG_2_-RM26 (25 kBq, 45 pmol in 100 µL PBS per mouse) and were euthanized 3 and 24 h pi.

Specificity of ^57^Co-DOTA-PEG_2_-RM26 to GRPR was tested in NMRI mice 1 h pi of 45 or 20 nmol of labeled conjugate (25 kBq, in 100 µL PBS per mouse).

Whole body PET/CT scans were performed using a Siemens Inveon preclinical PET/SPECT/CT-scanner on PC-3 xenografted male NOD-SCID mice (in-bread). The mice were anesthetized with a mixture of 1.5–2% isoflurane and 100% oxygen and intravenously injected with 0.3 nmol (range: 0.24–0.32 nmol) ^55^Co-X-PEG_2_-RM26 (approx. 2.5 MBq). At 3 h and 24 h pi, the mice were anesthetized again using isoflurane and PET/CT scanned with PET acquisition times of 15 min. and 30 min., respectively. CT parameters were 2 bed positions, 360 projections in 360 degrees’ rotation, and bin 4. CT and PET images were co-registered and the CT-based attenuation corrected PET data was reconstructed using an OSEM3D/MAP algorithm (4 OSEM3D iterations, 16 MAP subsets, and 18 MAP iterations, target resolution 0.8 mm). PET and CT data were analyzed using Inveon Research Workplace (Siemens Healthcare) and presented as MIPs adjusted to display a color scale from 0 to the maximum tumor uptake value.

## Supplementary information

Supplementary Information
