# Supplementary material for: Selection of an optimal macrocyclic chelator improves the imaging of prostate cancer using cobalt-labeled GRPR antagonist RM26
Source: Sci Rep. 2019 Nov 19;9:17086. doi: 10.1038/s41598-019-52914-y (PMC6863848; doi:10.1038/s41598-019-52914-y)
Supplement: Supplementary file 1 — Supplementary Information [file 41598_2019_52914_MOESM1_ESM.pdf]

Supplementary Information

Selection of an optimal macrocyclic chelator improves the imaging of prostate cancer using cobalt-labeled GRPR antagonist RM26

Bogdan Mitran*[1], Helge Thisgaard*[2,3], Sara Rinne[1], Johan Hygum Dam[2,3], Frishta Azami[1], Vladimir Tolmachev[5], Anna Orlova*[1,4], Ulrika Rosenström*[1§]

1 Department of Medicinal Chemistry, Uppsala University, Sweden;

2 PET & Cyclotron Unit, Department of Nuclear Medicine, Odense University Hospital, Odense, Denmark;

3 Department of Clinical Research, University of Southern Denmark, Odense, Denmark;

4 Science for Life Laboratory, Department of Medicinal Chemistry, Uppsala University, Sweden;

5 Department of Immunology, Genetics and Pathology, Uppsala University, Sweden.

Supplementary table 1. Comparative biodistribution of ^{57}Co-X-PEG$_2$-RM26 (X= NOTA, NODAGA, DOTA, DOTAGA) in PC-3-xenografted BALB/c nu/nu mice at 3 h and 24 h pi.

	NOTA		NODAGA		DOTA		DOTAGA	
Organ	3h	24h	3h	24h	3h	24h	3h	24h
Blood	0.019±0.004^{a,b}	0.006±0.001	0.032±0.009	0.006±0.003	0.028±0.006	0.007±0.002	0.02±0.01	0.005±0.002
Lung	0.065±0.005^{a}	0.09±0.15	0.05±0.01	0.02±0.01	0.06±0.01	0.023±0.004	0.07±0.03	0.02±0.02
Liver	0.070±0.004	0.025±0.005^{b}	0.08±0.02	0.02±0.01	0.078±0.009	0.034±0.003	0.08±0.02	0.04±0.01
Spleen	0.050±0.008^{b}	0.029±0.004^{a,c}	0.05±0.02^{b}	0.009±0.002^{b}	0.09±0.02	0.035±0.006^{c}	0.08±0.05	0.016±0.009
Pancreas	0.21±0.052^{b}	0.023±0.002^{b}	0.3±0.2^{b}	0.02±0.03	2.5±0.8^{c}	0.05±0.01^{c}	0.4±0.1	0.028±0.009
Stomach	0.3±0.1^{b}	0.05±0.03	0.39±0.09^{b}	0.04±0.01	2.1±0.6^{c}	0.06±0.02^{c}	0.4±0.3	0.018±0.004
Sm. Int.	0.098±0.028	0.06±0.03^{a,c}	0.3±0.3^{b}	0.015±0.007^{b}	0.9±0.1^{c}	0.06±0.02^{c}	0.13±0.05	0.02±0.01
Kidneys	0.93±0.05^{a,b,c}	0.09±0.03^{a,b,c}	2.5±0.8	0.7±0.4^{c}	1.9±0.3^{c}	0.8±0.2^{c}	3.5±0.4	1.8±0.5
Tumor	7.3±1.3	2.1±0.2^{b}	6.3±1.2^{b}	2.3±0.9^{b}	8.1±0.6	4.4±1.3^{c}	6.9±2.7	2.3±0.7
Muscle	0.08±0.10	0.011±0.005	0.02±0.01	0.006±0.005	0.4±0.7	0.02±0.01	0.04±0.01	0.007±0.007
Bone	0.04±0.04^{c}	0.08±0.05	0.1±0.2	0.007±0.002	0.08±0.03^{c}	0.09±0.06	0.020±0.005	0.02±0.03
GI	0.6±0.1^{a,b}	0.09±0.03^{a,b}	5±1^{c}	0.3±0.2	4.8±0.1^{c}	0.21±0.09	1.0±0.5	0.1±0.1
Body	0.6±0.4^{a,c}	0.18±0.06	1.3±0.3	0.1±0.1	0.9±0.4	0.2±0.1	1.9±0.9	0.3±0.3

The organ uptake values are expressed as a percentage of injected dose per gram of tissue (%ID/g), except for GI and carcass for which the values are expressed as a percentage of the injected dose per sample (%ID). Data were analyzed using GraphPad Prism (version 7.03, GraphPad Software Inc.) to determine significant statistical differences ($p<0.05$) by one-way ANOVA with Bonferroni correction for multiple comparisons.

Significant difference ($p<0.05$) at the same time point.

a. *Significantly different from NODAGA*
b. *Significantly different from DOTA*
c. *Significantly different from DOTAGA*

Supplementary table 2. Tumor-to-normal-tissues ratios of ^{57}Co-X-PEG_2-RM26 (X= NOTA, NODAGA, DOTA, DOTAGA) in PC-3-xenografted BALB/c nu/nu mice at 3 h and 24 h pi.

	NOTA		NODAGA		DOTA		DOTAGA	
Organ	3h	24h	3h	24h	3h	24h	3h	24h
Blood	378±26 [a,b,c]	350±49[b]	203±32[b,c]	406±70[b]	294±45	598±70	289±58	488±255
Lung	114±28	87±56[b]	135±12	212±136	142±28	192±36	119±65	197±83
Liver	105±20	86±21	80±8[b]	95±12	105±8	128±30[c]	91±25	67±20
Spleen	145±20[b]	74±11[a,b,c]	134±33	269±157	97±21	125±25	112±54	156±51
Pancreas	37±14[b]	92±12	25±11[b]	249±213	3.5±0.8[c]	89±5	20±9	84±17
Stomach	29±10[a,b]	61±37	16±1[b]	72±35	4±1[c]	71±19	20±9	136±62
Sm. Int.	79±26[b]	37±10[a,b]	41±34	165±70[b]	9±2[c]	75±21	59±31	235±289
Kidneys	8±1[a,b,c]	23±6[a,b,c]	2.6±0.5[b]	4±1[b,c]	4.3±0.7[c]	5±0.6[c]	1.9±0.5	1.3±0.4
Muscle	232±197	217±88	353±157	492±231	260±177	340±217	191±95	468±216
Bone	115±90[c]	38±19[a]	179±124	378±267	112±40[c]	61±35	335±75	138±94

Data were analyzed using GraphPad Prism (version 7.03, GraphPad Software Inc.) to determine significant statistical differences ($p<0.05$) by one-way ANOVA with Bonferroni correction for multiple comparisons. Significant difference ($p<0.05$) at the same time point.

a. *Significantly different from NODAGA*
b. *Significantly different from DOTA*
c. *Significantly different from DOTAGA*

Supplementary table 3. Comparison of tumor-to-normal-tissues ratios for radiolabeled BN-based GRPR antagonist in PC-3-xenografted mice at 24 h pi.

Organ	PET					SPECT			
	**Co-DOTA-PEG2-RM26**	Cu-NOTA-MJ9 [c]	Cu-NODAGA-MJ9 [c]	Cu-TE2A-PEG4-RM26 [d]	Co-NOTA-AMBA [b,e]	In-NODAGA-PEG2-RM26 [f]	In-DOTA-RM2 [g]	In-DOTA-RM26 [h]	In-DOTA-AMBA [b, h]
Blood	**598±70**	ND [a]	ND	255±35	150	253±93	2 280	658	147
Liver	**128±30**	5	5	5.4±1.1	35	6.6±0.2	76.0	34.6	21.1
Pancreas	**89±5**	32	20	117±19	<1	12±2	21.5	44	0.06
Intestine	**75±21**	ND	ND	ND	1	27±5	170	164.5	1.6
Kidneys	**5±0.6**	8	5	17±4.2	4.5	1.1±0.2	5.5	6.5	2.3
Muscle	**340±217**	40	40	482±151	ND	646±408	342	219	21.1
Bone	**61±35**	ND	ND	ND	ND	104±92	34	47	12

a No data was provided

b GRPR agonist

c Gourni E, Mansi R, Jamous M, Waser B, Smerling C, Burian A, Buchegger F, Reubi JC, Maecke HR. N-terminal modifications improve the receptor affinity and pharmacokinetics of radiolabeled peptidic gastrin-releasing peptide receptor antagonists: examples of 68Ga- and 64Cu-labeled peptides for PET imaging. J Nucl Med. 2014;55(10):1719-25.

d Gourni E, Del Pozzo L, Kheirallah E, Smerling C, Waser B, Reubi JC, Paterson BM, Donnelly PS, Meyer PT, Maecke HR. Copper-64 Labeled Macrobicyclic Sarcophagine Coupled to a GRP Receptor Antagonist Shows Great Promise for PET Imaging of Prostate Cancer. Mol Pharm. 2015;12(8):2781-90.

e Dam JH., Olsen BB, Baun C, Høilund-Carlsen PF, Thisgaard H. In Vivo Evaluation of a Bombesin Analogue Labeled with Ga-68 and Co-55/57. Mol. Imaging Biol. 2016; 18, 368-76.

f Mitran B, Varasteh Z, Selvaraju R, Lindeberg G, Sörensen J, Larhed M, Tolmachev V, Rosenström U, Orlova A. Selection of Optimal Chelator Improves the Contrast of GRPR Imaging Using Bombesin Analogue RM26. Int J Oncol. 2016;48(5):2124-34.

g Mansi R, Wang X, Forrer F, Waser B, Cescato R, Graham K, Borkowski S, Reubi JC, Maecke HR. Development of a potent DOTA-conjugated bombesin antagonist for targeting GRPr-positive tumours. Eur J Nucl Med Mol Imaging 2011; 38:97–107.

h Mansi R, Wang X, Forrer F, Kneifel S, Tamma ML, Waser B, Cescato R, Reubi JC, Maecke HR. Evaluation of a 1,4,7,10-Tetraazacyclododecane-1,4,7,10-Tetraacetic Acid–Conjugated Bombesin-Based Radioantagonist for the Labeling with Single-Photon Emission Computed Tomography, Positron Emission Tomography, and Therapeutic Radionuclides. Clin Cancer Res 2009;15(16) 5240-49.

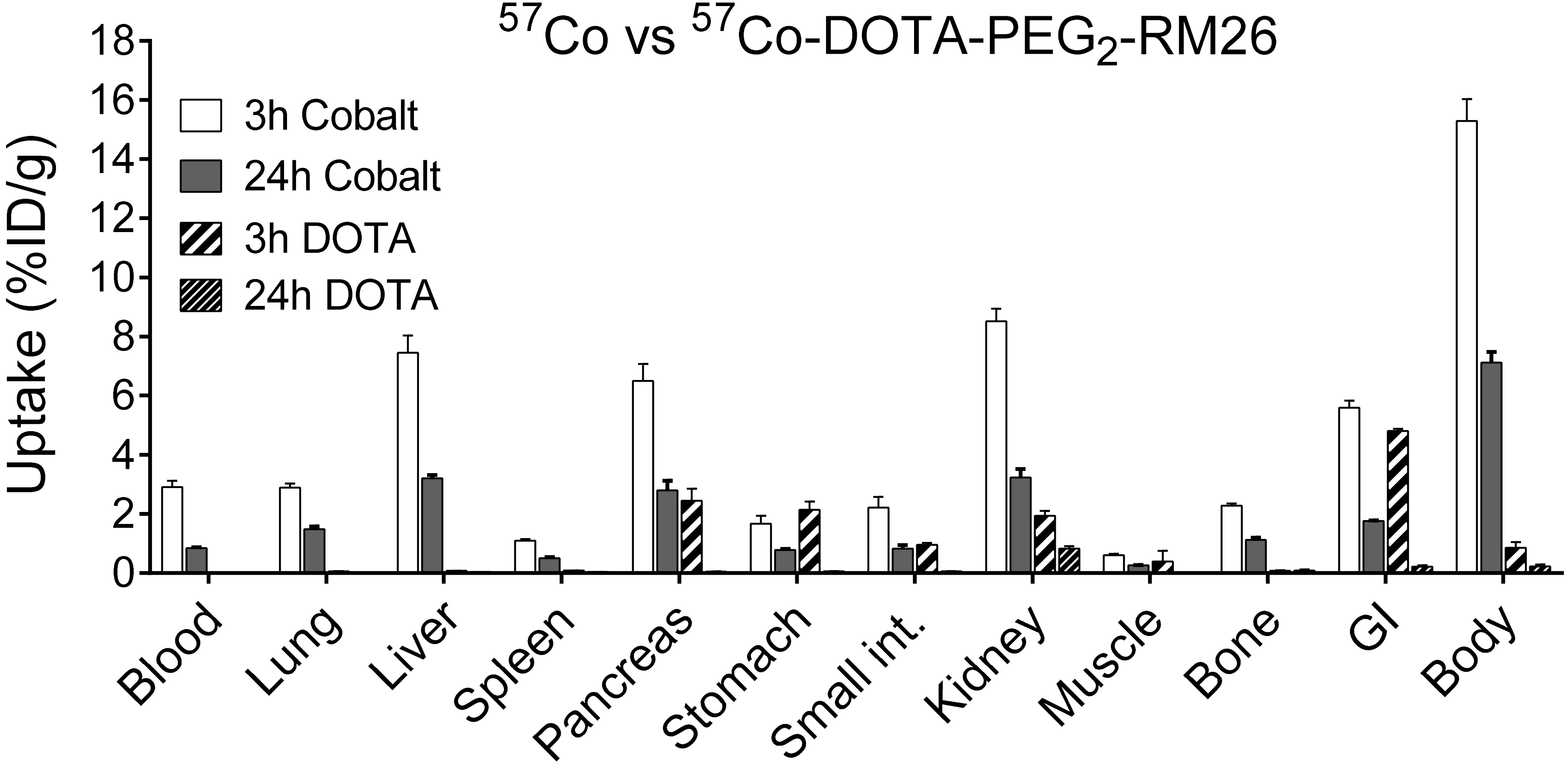

Supplementary figure 2. Biodistribution of [^{57}Co]$CoCl_2$ in NMRI mice 3 and 24 h pi. Data for biodistribution of ^{57}Co-DOTA-PEG_2-RM26 are given for comparison.

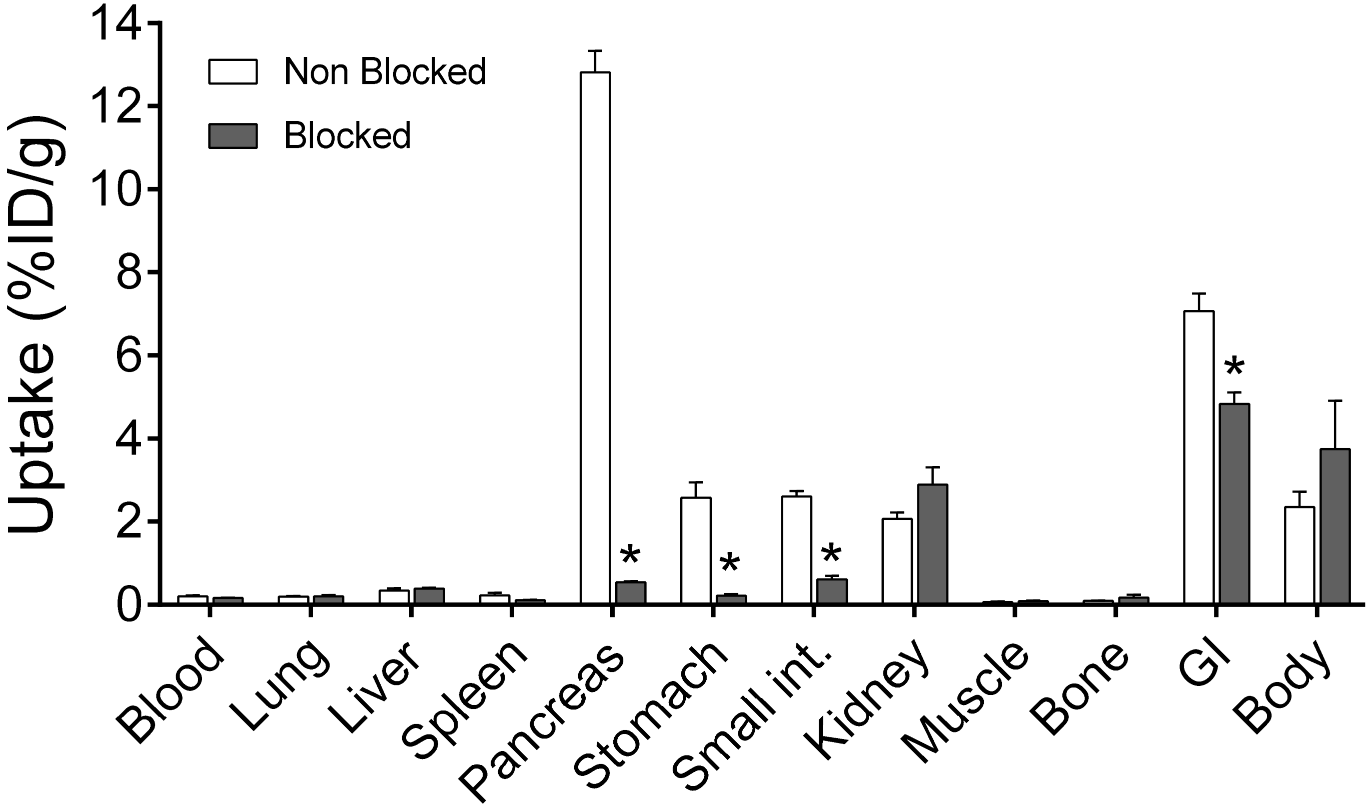

Supplementary figure 1. Biodistribution of ^{57}Co-DOTA-PEG$_2$-RM26 1 h pi of 45 (Non Blocked) or 20 nmol in NMRI mice. Data were analyzed using GraphPad Prism (version 7.03, GraphPad Software Inc.) to determine significant statistical differences ($p<0.05$) by an unpaired, two-tailed t-test.
